# Development and implementation of the frog-in-maze game to study upper limb movement in people with Parkinson’s disease

**DOI:** 10.1038/s41598-023-49382-w

**Published:** 2023-12-20

**Authors:** Tuhin Virmani, Aaron S. Kemp, Lakshmi Pillai, Aliyah Glover, Horace Spencer, Linda Larson-Prior

**Affiliations:** 1 Department of Biomedical Informatics, University of Arkansas for Medical Sciences, 4301 W. Markham St., #500, Little Rock, AR 72205 USA; 2 Department of Neurology, University of Arkansas for Medical Sciences, 4301 W. Markham St., #500, Little Rock, AR 72205 USA; 3 Department of Biostatistics, University of Arkansas for Medical Sciences, 4301 W. Markham St., #500, Little Rock, AR 72205 USA; 4 Department of Neurobiology, University of Arkansas for Medical Sciences, 4301 W. Markham St., #500, Little Rock, AR 72205 USA

**Keywords:** Parkinson's disease, Predictive markers

## Abstract

Upper-limb bradykinesia occurs early in Parkinson’s disease (PD) and bradykinesia is required for diagnosis. Our goal was to develop, implement and validate a game “walking” a frog through a maze using bimanual, alternating finger-tapping movements to provide a salient, objective, and remotely monitorable method of tracking disease progression and response to therapy in PD. Twenty-five people with PD and 16 people without PD participated. Responses on 5 different mazes were quantified and compared to spatiotemporal gait parameters and standard disease metrics in these participants. Intertap interval (ITI) on maze 2 & 3, which included turns, was strongly inversely related to stride-length and stride-velocity and directly related to motor UPDRS scores. Levodopa decreased ITI, except in maze 4. PD participants with freezing of gait had longer ITI on all mazes. The responses quantified on maze 2 & 3 were related to disease severity and gait stride-length, were levodopa responsive, and were worse in people with freezing of gait, suggesting that these mazes could be used to quantify motor dysfunction in PD. Programming our frog-in-maze game onto a remotely distributable platform could provide a tool to monitor disease progression and therapeutic response in people with PD, including during clinical trials.

## Introduction

Upper limb movements have been objectively shown to be affected in early untreated Parkinson’s disease (PD)^1^, and quantitative finger tapping velocities have been inversely correlated to disease severity^2^. Bradykinesia, the slowing and tapering in amplitude on repetitive movements of the hands and feet is required for a clinical diagnosis based on both the UK brain bank^3^ and the newer MDS criteria^4^. Upper limb bradykinesia impacts performance of bimanual tasks such as cutting meat, buttoning clothes and shampooing one’s hair^5^, leading to decreased quality of life. As the PD progresses, a complete breakdown in motor function can occur in the form of episodic, paroxysmal motor arrests (or freezes), during repetitive movements such as handwriting, speech, and gait^6–9^. The risk and prevalence of these freezes also increases with longer disease duration^6,8,10,11^. Upper limb freezing may also precede development of freezing of gait (FOG)^12^.

Repetitive tapping tasks have been used to study bradykinesia in PD^1,13^, have been used to evaluate upper limb freezing^14,15^, and have demonstrated the sequence effect^16^ which manifests as a decrement in amplitude that precedes freezing episodes. We previously showed that tap amplitude on a bimanual finger tapping task was correlated to gait stride-length in individuals with PD in the levodopa OFF state^17^. Tap amplitude on this task also improved with levodopa proportionally to the improvement seen in stride-length with levodopa. Tasks that involve finger tapping may therefore be appropriate tools to measure motor disease function.

Multiple groups have developed mobile aps that include measurement of bradykinesia using finger tapping behaviors including the Roche PD Mobile Application^18^, the CloudUPDRS Parkinson’s study^19^ and the mPOWER study^20^. Tapping results from the mPOWER dataset have shown utility in splitting people with PD into groups based on disease severity^21^, and amongst the 5 features collected was the best classifier for the PD group^20^.

One group has developed a game called the “goalkeeper game” to assess motor and cognitive performance in PD^22^. By tapping on three arrow keys on a keyboard the “player” controls the goalkeeper during a penalty shootout in the game of soccer (or football). The authors found that the memory components of the variables extracted from their game correlated better with the results on the Dynamic Gait Index (DGI) test than scores on the Montreal Cognitive Assessment (MoCA).

The development of techniques to monitor upper limb function inexpensively in a home-based setting using technology present in most homes will improve ease of monitoring long term disease progression, response to medication clinically and also possibly improve outcome monitoring in clinical trials^23^. While simple tapping behaviors provide output of simple motor function, developing a task that integrates cognitive and motor tasks, as was the case for the goalkeeper game^22^, and remains easy to perform in a population of older adults like people with PD and other neurodegenerative disease, would be very beneficial. To this end, we designed a game that utilized bimanual finger tapping to “walk” a frog through a maze. The goal was to record bimanual movement that could simulate bipedal gait and allow for coding of obstacles that are encountered during walking. To determine our games’ efficacy as an effective marker for PD function, we tested the game on people with and without PD. We also determined whether quantified measures obtained from the game reflected disease severity and were responsive to dopaminergic therapy.

## Methods

### Study population

Participants with PD based on UK brain bank diagnostic criteria^3^ and age-matched adults (controls) between the ages of 45–90 were recruited from the Movement Disorders Clinic (MDC) at the University of Arkansas for Medical Sciences (UAMS) between February-November 2019. 51 participants were enrolled (24 controls, 27 PD) and analyzed. Exclusion criteria comprised inability to walk on the Zeno walkway, falls > 1/day, cognitive impairment sufficient to impair capacity for informed consent, diagnosis of a neurologic disorder (other than PD for the PD group), diagnosis of a psychiatric disorder other than those associated with PD, the use of anti-dopaminergic medications in the year prior to enrollment, chronic back, hip or knee pain that was not controlled, severe osteoarthritis, hip or knee replacement surgery or spine surgery in the last 12 months or complicated by persistent pain, and inability to complete questionnaires in English. The study was approved by the UAMS institutional review board (UAMS IRB# 228861), and written informed consent was obtained from all participants prior to study assessments being performed. The study was conducted in accordance with the guidelines of the Declaration of Helsinki.

### Standard motor and non-motor assessments

A complete Unified Parkinson’s Disease Rating Scale (UPDRS)^24^ was performed as the gold standard measure of PD function. The New Freezing of Gait Questionnaire was used to classify the PD participants according to the presence or absence of freezing of gait (FOG) based on an Item 1 score of 1 or 0 respectively. The Montreal Cognitive Assessment (MoCA)^25^ was used as a cognitive screening score, and the Hamilton Depression (HAM-D)^26^ and Anxiety (HAM-A)^27^ rating scales were used to assess for depression and anxiety in all participants.

### Gait assessments

Participants were instructed to walk at a “comfortable” pace, 8 lengths of a 20′ × 4′ instrumented gait mat (Zeno Walkway, Protokinetics, Haverton, PA), and data were collected and analyzed using the Protokinetics movement analysis software (PKMAS)^28,29^. Mean stride-length, stride-time, stride-width and stride-velocity were extracted for each individual’s continuous gait during straight walking using the intrinsic algorithms of PKMAS as in prior published studies^30,31^.

### Frog-in-maze game

**Figure 1:**
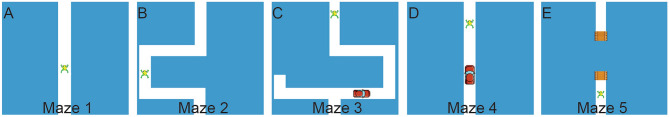
Frog-in-maze game. Mazes used in the implementation of this project. Mazes simulating (**A**) movement or “walking” the frog in a straight line, (**B**) forward movement interrupted by requirement to turn either left or right, (**C**) straight and turning movement with additional time pressure of a car chasing behind the frog, (**D**) straight movement with time pressure, and (**E**) a stop-start movement simulated by building a bridge to advance.

The game was developed using E-Prime v3 (Psychology Software Tools, Pittsburg, PA) to present a continuous sequence of stimuli which depicts a “frog” icon advancing or “walking” along a path from the bottom of the screen to the top. Using two button boxes (Current Designs, Philadelphia, PA) placed below their right and left hands, participants “walked” the frog through the mazes. Seated comfortably at a table, participants were instructed to bimanually and alternately tap the inner buttons on each button box with the right AND left index finger to move the frog forward in the maze, and to use the right OR left index AND middle fingers in alternating unimanual taps to move the frog right or left, respectively. The program was configured to record the time elapsed between each successful advancement of the frog along the path. If a participant performed an incorrect sequence of taps using the wrong fingers or the wrong sequence of finger movements for a given move, the program did not move the frog, and did not log a response until a successful sequence of movements led to advancement of the frog. Thus “incorrect” responses would increase that specific intertap interval. The five mazes were designed to include a straight path (Fig. 1A), turns (Fig. 1B,C), a car chase (Fig. 1C,D) and a stop-and-go task (Fig. 1E), as turning, time pressure and gait initiation provoke a clinical gait phenomena called freezing of gait^32,33^. Participants performed the mazes in sequential order from 1 to 5 due to the programming limitations of E-Prime.

### Dopaminergic response

All PD participants underwent UPDRS, gait assessments and played the frog-in-maze game in the morning in their effective levodopa OFF-state after withholding their Parkinson’s medications overnight as per prior protocols (PD-OFF)^15,34^. Comparisons to controls and associations were performed in the OFF-levodopa, or relative levodopa unmedicated state in order to compare the underlying disease state without the potential symptomatic benefit from levodopa. PD participants who were taking levodopa (n = 22) as part of their clinical regimen were examined again 60 min after their regular morning dose of levodopa (PD-ON) in order to determine if amplitude setting in the upper limb and gait both responded similarly to levodopa in individual participants. Participants were not excluded from participating if they were not clinically treated with levodopa as a portion of the analysis was performed using the levodopa OFF-state data only.

### Statistical analysis

Statistical analysis was performed using SPSS 24 (IBM). A Mann–Whitney test was used to compare the control and PD group differences given the distribution of the data was non-parametric on normality testing. To account for non-normal data, natural log transforms of Maze ITI, gait, motor and non-motor assessments were used for linear regression analysis. Kendall’s tau-b correlation coefficients were also used to compare these different assessments. A post-hoc Benjamini–Hochberg (BH) adjustment was applied to account for the multiple comparisons for each specific sub-analysis. For example, for spatiotemporal gait parameters 20 comparisons were adjusted for (5 mazes × 4 measures). Linear regression analysis appeared to be more conservative in the number of variables showing significant results and therefore these are reported as the primary results in the manuscript. However for comparison Kendall’s tau-b results are also provided in the supplementary figures. Levodopa responsiveness (OFF to ON dopaminergic medications) of the intertap interval on the different mazes in the PD group was determined using Wilcoxon signed rank test. Subgroup analysis of noFOG and FOG participants was performed using the Mann–Whitney test to determine if there were group differences on maze response in either the OFF-levodopa or ON-levodopa conditions. The Wilcoxon signed-rank test was also performed independently in each subgroup to determine levodopa response (ON/OFF levodopa) in the different mazes.

## Results

### Participant populations

Both PD and control populations were well matched for age (Table 1). The ratio of female to male participants was significantly higher in the control group (chi-square *p* = 0.005). PD participants had a mean disease duration of 9.2 ± 5.8 years and 32% had a freezing of gait phenotype (FOG). Cognitive scores were similar between groups but the PD group had higher anxiety, depression and RBD-Q scores. While some of the controls had low scores on the UPDRS, none of them had clinical Parkinsonism based on history or met criteria for Parkinson’s disease based on examination by a movement disorders trained neurologist (TV).

**Table 1:** Demographics and clinical rating scores of participants.

	Controls (n = 16)	Parkinson’s disease (n = 25)
Age (years)	65.9 ± 7.0	68.6 ± 8.0
Sex (Female/male)	11/5	6/19^#^
Disease duration (years)	–	9.2 ± 5.8
Motor UPDRS Score	3.9 ± 3.2	23.8 ± 11.5*
Total UPDRS Score	6.7 ± 4.9	39.4 ± 19.2*
Percent w/ FOG	–	32% (n = 8)
New FOG-Q score (in those with FOG)	–	15.9 ± 6.3 (n = 8)
MoCA Score	27.4 ± 1.8	25.6 ± 3.7
Hamilton anxiety scale score	2.4 ± 2.3	6.1 ± 5.8*
Hamilton depression scale score	2.8 ± 2.5	6.7 ± 4.8*
REM sleep behavior disorder questionnaire score	2.3 ± 1.6	5.9 ± 3.0*
On levodopa	–	92% (n = 22)
LDopa Dose (mg/day)	–	779 ± 448
Duration on Ldopa (years)	–	6.3 ± 4.8
On Dopamine agonist	–	13% (n = 10)
On MAO-I	–	30% (n = 24)
LEDD	–	801 ± 459

Values are mean ± standard deviation.

*p < 0.05 on Mann–Whitney test.

^#^*p* < 0.05 on Chi-square test.

### Parkinson’s disease versus control participant responses on the frog-in-maze game

The PD groups response in the levodopa OFF or unmedicated state (PD-OFF) was compared with the control participants (Table 2). While the mean response times were longer in the PD-OFF state, they were not statistically significant due to the high inter-participant variability in performance. As there was a difference in the ratio of males to females between the PD and control groups, we also analyzed the response on each maze based on participant sex and found no differences (Supplementary Table S1). We therefore performed subsequent analysis for the whole group independent of participant sex.

**Table 2:** Frog-in-maze mean intertap intervals in controls and people with Parkinson’s disease in the OFF-levodopa state.

	Control (n = 16)	PD-OFF (n = 25)
Maze 1 Intertap interval (ms)	150 ± 50	182 ± 104
Maze 2 Intertap interval (ms)	356 ± 247	462 ± 302
Maze 3 Intertap interval (ms)	232 ± 122	310 ± 230
Maze 4 Intertap interval (ms)	126 ± 50	166 ± 155
Maze 5 Intertap interval (ms)	165 ± 71	204 ± 157

All values are reported as mean ± standard deviation.

### Frog-in-maze game versus objective spatiotemporal gait measures

**Figure 2:**
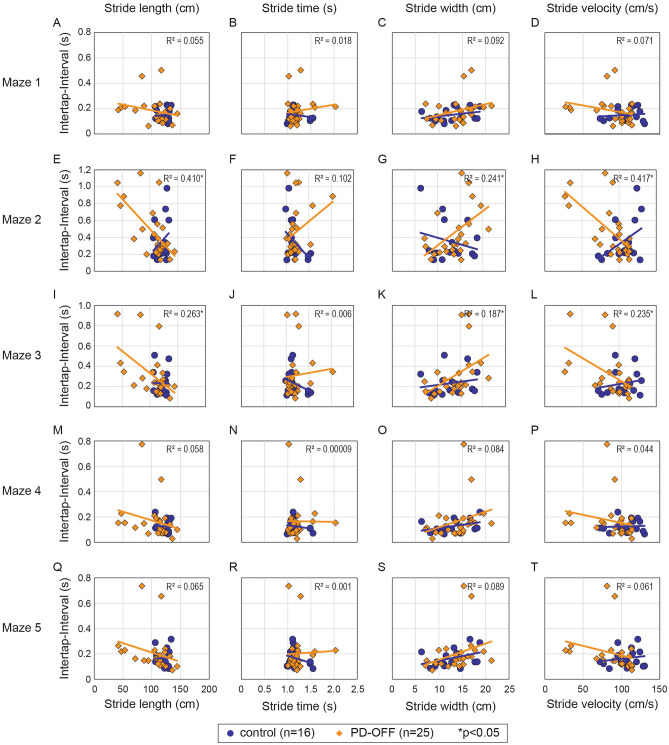
Maze responses compared to spatiotemporal gait in the levodopa OFF-state. Box plots of the each mazes intertap interval plotted against gait stride length, stride time, stride width and stride velocity on (**A**–**D**) maze 1, (**E–H**) Maze 2, (**I**–**L**) Maze 3, (**M**–**P**) Maze 4, and (**Q**–**T**) Maze 5. Orange diamonds are individual PD patients responses in the OFF levodopa state (n = 25) and blue circles are non-PD participants (n = 16). R^2^ values denote results of linear regression analysis for the PD participants, and * denotes statistically significant results.

Inter-tap interval in each maze as a function of spatiotemporal gait parameters is plotted in Fig. 2 for each individual. To account for non-normal distribution of the data, natural log transforms of the ITI and gait parameter values were used for linear regression analysis. In the PD-OFF state longer ITI on Maze 2 & 3 were significantly related to shorter stride length (Fig. 2E,I) and slower stride-velocity (Fig. 2H,L) and on Maze 3 with longer stride-width (Fig. 2K) after BH correction for 20 comparisons. However, important associations based on unadjusted results were also found for Maze 2, 4, 5 ITI and longer stride width (Fig. 2G,O,S, respectively) (Supplementary Table S2). Results were not significant for other comparisons (Fig. 2A-D,F,J,M,N,P–R). Kendall’s tau-b correlation coefficients reproduced these associations (Supplementary Table S2), but linear regression analysis was more conservative in statistically significant results and therefore used as primary analysis with Kendall’s tau-b results also reported in the supplementary tables (Supplementary Table S2).

Using linear regression analysis of natural log converted ITI and UPDRS scores, Maze 2–5 ITI were also related to motor UPDRS scores and Maze 2 to total UPDRS scores after application of a BH correction for 10 measures (5 mazes × 2 assessments) (Supplementary Table S3).

### Frog-in-maze game versus non-motor assessments

In PD participants, comparing the non-motor assessments performed with the PD-OFF intertap interval on the mazes using linear regression of the converted values, none of the assessments showed significant relationship. However, the relationship between MoCA scores with Maze 2 ITI (R^2^ = 0.142, *p* = 0.064) were near significance and with Maze 3 ITI (R^2^ = 0.176, *p* = 0.037) were significant based on unadjusted results (Supplementary Table S4).

### Frog-in-maze levodopa response

**Figure 3:**
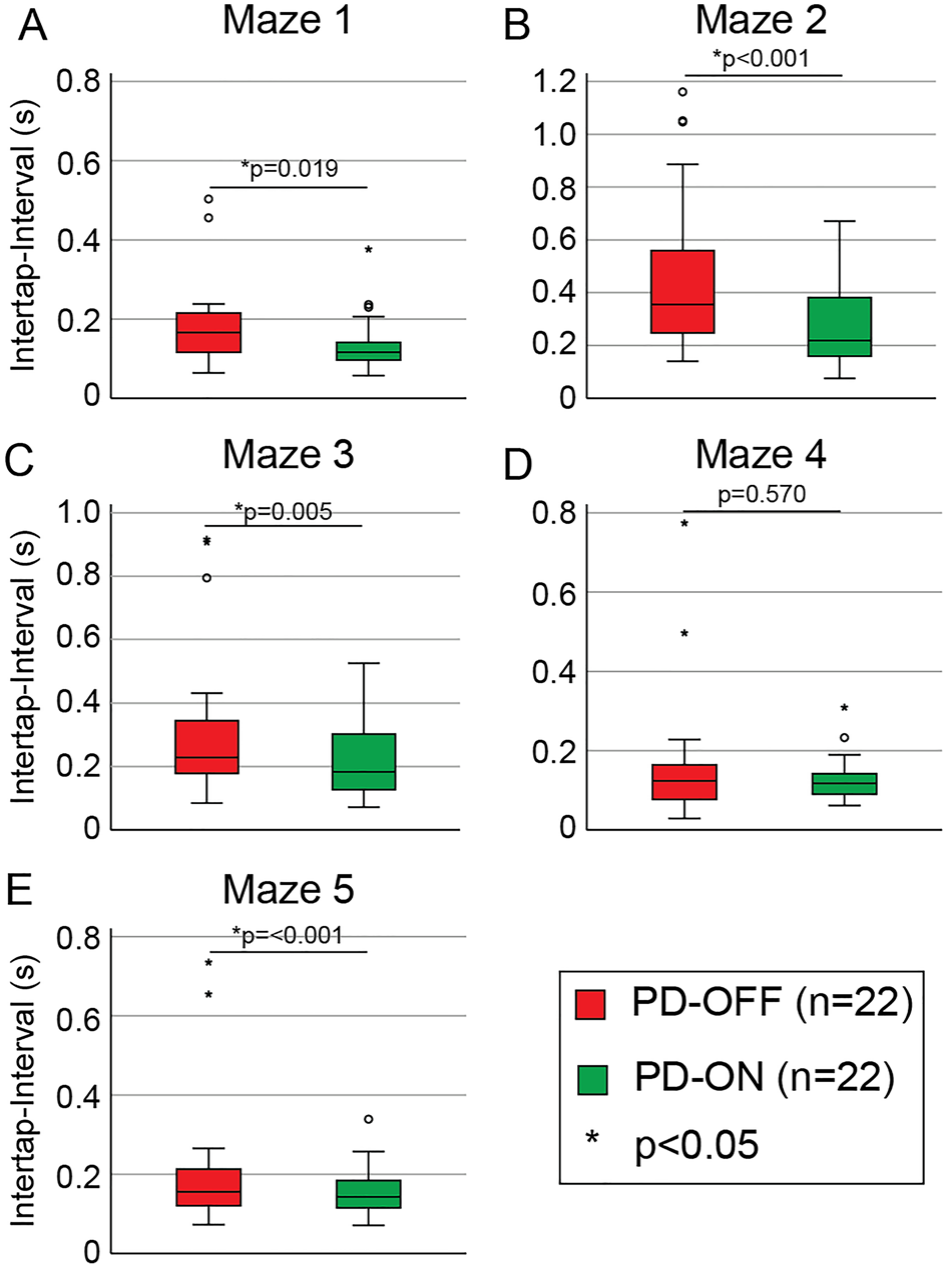
Levodopa response of Maze responses. (**A**–**E**) Box-plots of each mazes intertap interval in twenty-two PD participants in the levodopa OFF-state (red bars) and levodopa-ON state on mazes 1–5 respectively. Significant improvement in intertap interval was seen on Maze 2 and 3.

Twenty-two PD participants repeated the frog-in-maze assessment approximately 1 h after their regular morning dose of levodopa and the intertap interval ON and OFF levodopa are shown in Fig. 3. The inter-tap interval was significantly faster ON levodopa compared to OFF levodopa during performance of all mazes (Fig. 3A–C,E) except Maze 4 (Fig. 3D) using a Wilcoxon signed rank test (Supplementary Table S5).

We also compared the change (delta) in ITI before and after levodopa on each maze to the delta in motor and total UPDRS scores for each individual using linear regression analysis of the natural log transformed values. Delta ITI on all except Maze 1 were related to delta motor and total UPDRS scores after a BH correction for 10 comparisons (5 mazes × 2 assessments)(Supplementary Table S6).

### Subgroup analysis of gait freezers versus non-freezers on the frog-in-maze game

**Figure 4:**
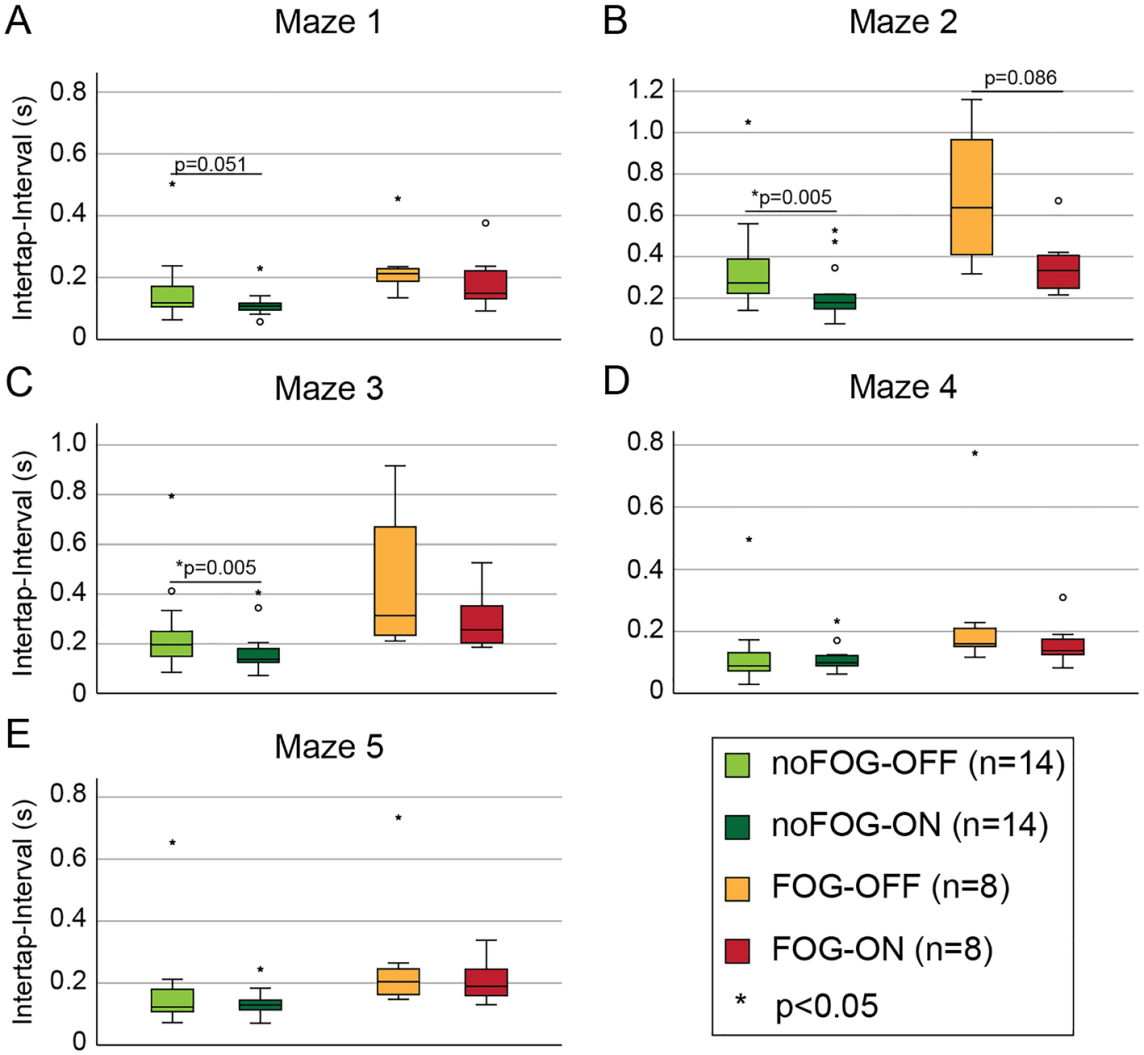
Subgroup analysis of PD gait freezers and non-freezers. (**A**–**E**) Box-plots of intertap interval for each maze for PD freezers and non-freezers in the levodopa OFF-state and ON-state. There were significant group differences between freezers and non-freezers in the OFF-levodopa and ON-levodopa states. Overall group differences were most prominent on Maze 2 independent of levodopa state. Improvement in intertap interval was significant in non-freezers on maze 2 and 3 and in freezers on maze 2.

PD participants were split into subgroups based on the presence (FOG group n = 8) or absence (noFOG group n = 14) of freezing of gait and their respective intertap intervals on each maze are plotted in Fig. 4 in the OFF levodopa and ON levodopa conditions. NoFOG participants had significantly faster ITI on all 5 mazes compared to FOG participants in both the levodopa ON- and OFF-states (Figure Supplementary Table S7). The noFOG group had faster intertap interval in the ON compared to OFF levodopa state on Mazes 2 (Fig. 4B) and 3 (Fig. 4C) with a BH correction for 5 comparisons (5 mazes)(Supplementary Table S8). However, in the FOG group there was a levodopa response on Maze 2 only based on unadjusted results (Fig. 4B; Supplementary Table S8). Maze 1, 4 and 5 (Fig. 4A,D,E) did not show significant levodopa responses in the sub groups.

### Frog-in-maze game versus gait, motor and non-motor features in the Levodopa ON state

**Figure 5:**
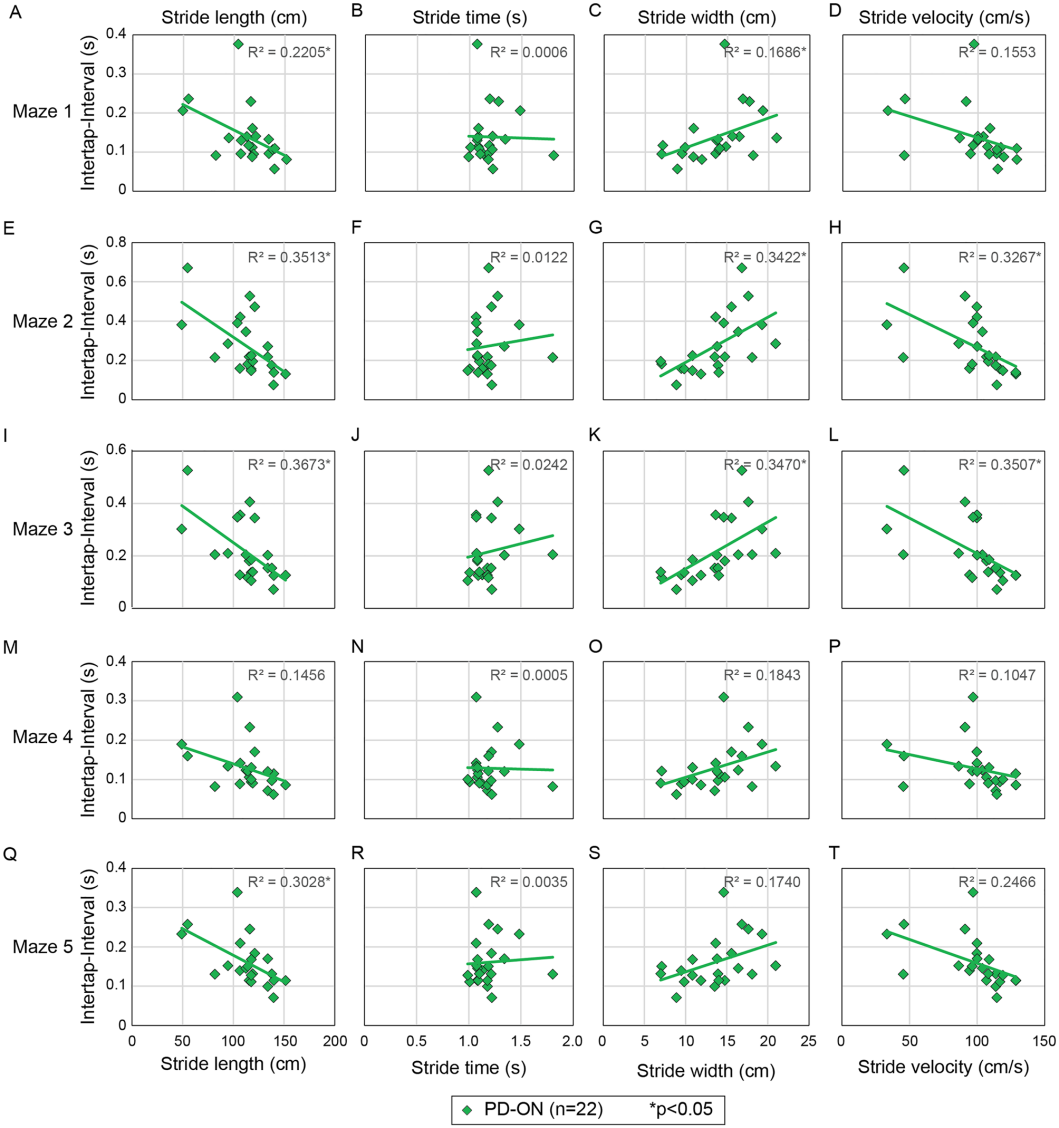
Maze responses compared to spatiotemporal gait in the levodopa ON-state. Box plots of the each mazes intertap interval plotted against gait stride length, stride time, stride width and stride velocity on (**A**–**D**) maze 1, (**E–H**) Maze 2, (**I**–**L**) Maze 3, (**M**–**P**) Maze 4, and (**Q**–**T**) Maze 5. Orange diamonds are individual PD participant responses in the ON levodopa state (n = 22). R^2^ values denote results of linear regression analysis for the PD participants, and * denotes statistically significant results.

To evaluate the utility of our game in day-to-day clinical use when people with PD typically present to clinic in the levodopa ON or medicated state (PD-ON; n = 22), we also compared the ON levodopa ITI results on the mazes and the gait, motor and non-motor assessments. In the PD-ON state longer ITI was significantly related to shorter stride length on Maze 1–3 & 5 (Fig. 5A,E,I,Q), slower stride velocity on Maze 2 & 3 (Fig. 5H,L) and wider stride width on Maze 1–3 (Fig. 5C,G,K) after BH correction for 20 comparisons (Supplementary Table S9). Results were not significant for other maze-gait comparisons (Fig. 5B,D,F,J,M–P,R–T)). PD-ON state ITI results were not related to ON-state motor or total UPDRS scores (Supplementary Table S10) or non-motor features (Supplementary Table S11) after BH correction. However, there was a relationship between Maze 2 & 3 ITI and motor and total UPDRS scores only based on unadjusted results (Supplementary Table S10).

## Discussion

In this study we designed a novel frog-in-maze game to objectively quantify finger tapping movements. Importantly, we demonstrate that the tapping tasks in our game can be performed by a population of older adults with and without neurodegenerative disease. We also show that in PD participants performing select mazes involving turning type behaviors, the intertap interval is related to the motor UPDRS score, that the tap interval is related to gait stride-length and stride-velocity, and that the tap interval improves with levodopa dosing. We also demonstrate that subgroups of people with PD with and without freezing of gait can be differentiated using Maze 2. As this game in its current form can be enhanced to allow distribution on multiple platforms, it could be used in home-monitoring of longitudinal disease progression and potentially as an objective surrogate marker in clinical trials of novel therapeutics.

Tapping behaviors have been demonstrated to be affected early in disease^1^, relate to disease severity^2,21^, and have been successfully deployed on mobile phone based platforms for real world data collection^18–20^. We have also previously shown in a simple tapping task, as in the more advanced game described in this work, that finger tapping amplitude relates to gait stride-length^17^. Our game adds to simple tapping tasks by incorporating a visual game that simulates gait by walking a frog through a maze environment using bimanual movements akin to bipedal gait. Additionally, real world obstacles such as turning the frog (maze 2 &3) helped differentiate subgroups of PD participants with more advanced gait phenotypes, i.e. the presence of freezing of gait. This is of special interest given that turning commonly provokes gait freezing in people with PD^35^ and also often is associated with falls in older adults that result in hip fractures^36^. The mazes that were developed to try and simulate time pressure (Maze 3&4) and stopping and then starting (Maze 5) did not show as significant a difference between PD participants with and without freezing although these are also reported to provoke gait freezing, but not as commonly as turning.

Our current paradigm however does not allow us to distinguish between two mechanisms, a visual input of the “turn” in the maze leading to the group differences in maze 2 & 3 as opposed to a motor set-shifting phenomena related to switching from bimanual to unimanual tapping to turn. During turning while walking, the motor plan shifts from more equal activation of both lower limbs to asymmetric activation of an outer leg circumducting around an inner pivoting leg. In our maze task to simulate this asymmetry, one hand stops moving while unimanual tapping occurs in the hand towards the side of the turn. Further tasks involving shifting from bimanual to unimanual tapping independent of the maze task along with a cognitive set-shifting task independent of motor output, could help differentiate between these two possibilities. Additionally, relationships to selective portions of a neuropsychiatric battery may also help further delve into cognitive mechanisms of the response. Nevertheless, as a task to distinguish PD severity our results suggest potential clinical utility independent of the exact mechanism.

Unlike a simple tapping task our game could also be developed into more advanced graphical interfaces that could quantify and track response to other everyday obstacles, including response to an intervention. Eventually incorporation of the game into a virtual reality (VR) environment, such as the cardboard google VR environment that works with a mobile phone, could allow development of tasks that include more visual, cognitive and motor integration. Such VR tasks are already used in studies aiming to enhance our understanding of the pathophysiologic mechanisms of gait disorders such as freezing of gait by simulating a walking environment in an fMRI scanner^37,38^.

Travel to in-person research visits is often a major limitation in a disease such as PD that limits not only mobility but cognitive function thereby impacting driving ability. Remote assessments could help improve clinical care and research participation in this population. We previously showed that even in a rural, medically underserved state in the United States (US) with relatively lower rates of cellular connectivity and internet access compared to the US mean, high quality clinical and research information can be reliably collected and analyzed from people with PD using telemedicine and can enable research enrollment from medically underserved areas^39^. Participant satisfaction in home-based care is also usually high^39–41^. Further development of our gaming environment could help provide a measure to successfully track disease progression in the home setting that may increase participation in research and improve care for people with PD.

One of the limitations of population-based studies with self-enrollment, referred to as Real World Data (RWD) collection, was demonstrated and acknowledged by the authors in the mPOWER study^20^. Their study design biased towards people who had access to an Apple smartphone, lived in the US and spoke English. This led to enrollment of younger individuals with higher income and education levels, especially in the non-PD control population^20^. Development of our game into a cross-platform product with instructions in multiple languages could help overcome this confounder. Additionally, electronic consents and shared IRBs have made it easier to perform multi-center studies; however, this still requires extensive administration at multiple sites and increased associated costs. Self-enrollment based population studies allow for rapid study enrollment but add the dimension of self-reported diagnostic status which cannot be verified. Staged validation of gaming environments may overcome these various barriers, moving from single-site to multi-site to self-enrollment based population studies. The use of shared database structures could also be implemented^42^.

One of the limitations of our study was that the program, by design, quantified the movement of the frog forward rather than quantifying individual finger taps. As a result, we could not distinguish between incorrect tapping sequences and prolonged time between tapping due, for example, to finger freezing. Additionally, the relatively short mazes did not provide adequate numbers of responses to differentiate responses at specific positions in a particular maze, such as the time of the turn, or the time when a stop-start behavior was required. Despite keeping the output quantification of the programming very basic at this time, we were still able to get disease relevant information. Future iterations will include more detailed response characterization which might provide further analytics of interest. The small number of participants was also a limitation and could be the reason the task did not significantly differentiate older adults without Parkinson’s disease from those with Parkinson’s disease as there was a high degree of heterogeneity in both groups. However, it appeared from subgroup analysis that the more severe phenotypes may have been driving the response. Positively however, maze 2 of the game still showed significant potential for further development with clear correlation in responses to disease severity and the ability to demonstrate levodopa response and display significantly slower responses in freezers compared to non-freezers. It is possible that larger populations would demonstrate differences on other mazes as well and warrants further exploration. The alternative to this however is that smaller samples can also yield higher associations which may not be seen in larger sample studies. External and larger validation samples are needed to confirm our findings on this task.

In summary we designed a game that simulated gait behaviors using bimanual finger tapping to move a frog through a maze. The maze included changes in patterned movement in response to a simulated turn that generated responses related to disease severity and worsened in people with the more severe gait phenotype of freezing of gait. The responses were also improved with levodopa suggesting dopaminergic pathways were being studied. Further development of our frog-in-maze game onto a platform that allows remote in-home access on multiple smartphone platforms could help improve the monitoring of disease progression and therapeutic response in people with PD, including during clinical therapeutic trials.

### Supplementary Information

Supplementary Information.

## Data Availability

The datasets generated during and analyzed during the current study are available from the corresponding author on reasonable request.
