# Supplementary material for: Development and implementation of the frog-in-maze game to study upper limb movement in people with Parkinson’s disease
Source: Sci Rep. 2023 Dec 20;13:22784. doi: 10.1038/s41598-023-49382-w (PMC10733393; doi:10.1038/s41598-023-49382-w)
Supplement: Supplementary file 1 — Supplementary Information. [file 41598_2023_49382_MOESM1_ESM.docx]

| Supplementary Table 1: Maze intertap interval (ITI) divided by group (PD or control) and sex (male or female) | | | | | | | | | | | | |
| --- | --- | --- | --- | --- | --- | --- | --- | --- | --- | --- | --- | --- |
|  | control | | | | | | PD | | | | | |
|  | female (n=11) | | male (n=5) | | Mann-Whitney | | female (n=6) | | male (n=19) | | Mann-Whitney | |
|  | mean | stdev | mean | stdev | Z | *p*-value | mean | stdev | mean | stdev | Z | *p*-value |
| Maze 1 | 144.4 | 50.3 | 163.8 | 54.5 | -0.85 | 0.40 | 228.3 | 123.9 | 167.5 | 96.1 | -1.59 | 0.11 |
| Maze 2 | 386.2 | 275.5 | 289.9 | 177.9 | -0.96 | 0.34 | 531.6 | 354.3 | 441.3 | 291.5 | -0.70 | 0.48 |
| Maze 3 | 229.7 | 122.3 | 236.2 | 137.0 | -0.17 | 0.87 | 341.2 | 289.0 | 300.7 | 216.2 | -0.19 | 0.85 |
| Maze 4 | 124.7 | 53.3 | 129.4 | 47.6 | -0.17 | 0.87 | 230.6 | 271.2 | 145.9 | 98.9 | -0.13 | 0.90 |
| Maze 5 | 161.0 | 65.5 | 176.6 | 89.2 | -0.51 | 0.61 | 244.2 | 244.4 | 190.7 | 124.0 | 0.00 | 1.00 |

| Supplementary Table 2: Natural log transformed linear regression, and Kendall’s tau-b models for Maze intertap intervals (ITI) compared to spatiotemporal gait measures in PD participants in the OFF levodopa state (n=25) | | | | | | | | | | | | |
| --- | --- | --- | --- | --- | --- | --- | --- | --- | --- | --- | --- | --- |
|  | Stride-length | | | | | | Stride-time | | | | | |
|  |  | | | | | |  | | | | | |
|  | Linear regression – natural log (ln) transform | | | Kendall’s tau-b | | | Linear regression – natural log (ln) transform | | | Kendall’s tau-b | | |
|  | R^2^ | *p* value | BH *p*  value | CC | *p* value | BH  *p* value | R^2^ | *p* value | BH *p*  value | CC | *p* value | BH  *p* value |
| Maze 1 ITI | 0.082 | 0.166 | 0.208 | -0.233 | 0.102 | 0.136 | 0.047 | 0.297 | 0.349 | 0.167 | 0.243 | 0.304 |
| Maze 2 ITI | **0.384** | **0.001** | **0.019** | **-0.393** | **0.006** | **0.023** | 0.097 | 0.130 | 0.217 | 0.167 | 0.243 | 0.286 |
| Maze 3 ITI | **0.295** | **0.005** | **0.033** | **-0.380** | **0.008** | **0.022** | 0.027 | 0.430 | 0.478 | 0.153 | 0.283 | 0.283 |
| Maze 4 ITI | 0.100 | 0.124 | 0.225 | -0.260 | 0.069 | 0.105 | 0.019 | 0.512 | 0.512 | 0.167 | 0.243 | 0.270 |
| Maze 5 ITI | 0.136 | 0.070 | 0.156 | **-0.393** | **0.006** | **0.020** | 0.024 | 0.464 | 0.488 | 0.167 | 0.243 | 0.256 |

| Supplementary Table 2 cont..: Natural log transformed linear regression, and Kendall’s tau-b models for Maze intertap intervals (ITI) compared to spatiotemporal gait measures in PD participants in the OFF levodopa state (n=25) | | | | | | | | | | | | |
| --- | --- | --- | --- | --- | --- | --- | --- | --- | --- | --- | --- | --- |
|  | Stride-width | | | | | | Stride-velocity | | | | | |
|  | Linear regression – natural log (ln) transform | | | Kendall’s tau-b | | | Linear regression – natural log (ln) transform | | | Kendall’s tau-b | | |
|  | R^2^ | *p* value | BH *p*  value | CC | *p* value | BH *p*  value | R^2^ | *p* value | BH *p*  value | CC | *p* value | BH  *p* value |
| Maze 1 ITI | 0.092 | 0.141 | 0.201 | 0.253 | 0.076 | 0.108 | 0.095 | 0.134 | 0.206 | -0.273 | 0.055 | 0.092 |
| Maze 2 ITI | 0.227 | 0.016 | 0.053 | **0.427** | **0.003** | **0.028** | **0.369** | **0.001** | **0.013** | **-0.407** | **0.004** | **0.022** |
| Maze 3 ITI | **0.266** | **0.008** | **0.040** | **0.413** | **0.004** | **0.025** | **0.245** | **0.012** | **0.048** | **-0.367** | **0.010** | **0.025** |
| Maze 4 ITI | 0.185 | 0.032 | 0.091 | **0.360** | **0.012** | **0.026** | 0.090 | 0.145 | 0.193 | -0.313 | 0.028 | 0.051 |
| Maze 5 ITI | 0.170 | 0.040 | 0.100 | **0.347** | **0.015** | **0.030** | 0.124 | 0.085 | 0.170 | **-0.460** | **0.001** | **0.025** |
| CC: correlation coefficient; BH: Benjamini-Hochberg corrected  Numbers in **bold** indicate significant p values after Benjamini-Hochberg (BH) correction | | | | | | | | | | | | |

| Supplementary Table 3: Natural log transformed linear regression, and Kendall’s tau-b models for Maze intertap intervals (ITI) compared to standard PD assessment scales in PD participants in the OFF levodopa state (n=25) | | | | | | | | | | | | |
| --- | --- | --- | --- | --- | --- | --- | --- | --- | --- | --- | --- | --- |
|  | Motor UPDRS | | | | | | Total UPDRS | | | | | |
|  | Linear regression – natural log (ln) transform | | | Kendall’s tau-b | | | Linear regression – natural log (ln) transform | | | Kendall’s tau-b | | |
|  | R^2^ | *p* value | BH *p*  value | CC | *p* value | BH *p*  value | R^2^ | *p* value | BH *p*  value | CC | *p* value | BH  *p* value |
| Maze 1 ITI | 0.138 | 0.067 | 0.074 | 0.165 | 0.252 | 0.252 | 0.076 | 0.181 | 0.181 | 0.188 | 0.190 | 0.212 |
| Maze 2 ITI | **0.308** | **0.004** | **0.040** | 0.292 | 0.042 | 0.099 | **0.227** | **0.009** | **0.040** | 0.295 | 0.040 | 0.099 |
| Maze 3 ITI | **0.278** | **0.007** | **0.040** | 0.286 | 0.047 | 0.099 | 0.166 | 0.043 | 0.061 | 0.262 | 0.068 | 0.099 |
| Maze 4 ITI | **0.261** | **0.009** | **0.040** | 0.319 | 0.026 | 0.088 | 0.147 | 0.059 | 0.074 | 0.255 | 0.076 | 0.099 |
| Maze 5 ITI | **0.243** | **0.007** | **0.040** | **0.434** | **0.003** | **0.026** | 0.174 | 0.038 | 0.063 | **0.376** | **0.009** | **0.044** |
| CC: correlation coefficient; BH: Benjamini-Hochberg corrected  Numbers in **bold** indicate significant p values after Benjamini-Hochberg (BH) correction | | | | | | | | | | | | |

| Supplementary Table 4: Natural log transformed linear regression, and Kendall’s tau-b models for Maze intertap intervals compared to non-motor assessment scales in the OFF-levodopa state (n=25) | | | | | | | | | | | | |
| --- | --- | --- | --- | --- | --- | --- | --- | --- | --- | --- | --- | --- |
|  | MoCA | | | | | | HAM-A | | | | | |
|  |  | | | | | |  | | | | | |
|  | Linear regression – natural log (ln) transform | | | Kendall’s tau-b | | | Linear regression – natural log (ln) transform | | | Kendall’s tau-b | | |
|  | R^2^ | *p* value | BH *p*  value | CC | *p* value | BH  *p* value | R^2^ | *p* value | BH *p*  value | CC | *p* value | BH  *p* value |
| Maze 1 ITI | 0.006 | 0.707 | 1.000 | -0.102 | 0.493 | 0.903 | 0.068 | 0.207 | 1.000 | -0.090 | 0.541 | 0.903 |
| Maze 2 ITI | 0.142 | 0.064 | 0.740 | -0.291 | 0.050 | 0.498 | 0.000 | 0.997 | 1.000 | -0.028 | 0.851 | 0.903 |
| Maze 3 ITI | 0.176 | 0.037 | 0.740 | -0.291 | 0.050 | 0.498 | 0.017 | 0.531 | 1.000 | -0.041 | 0.778 | 0.903 |
| Maze 4 ITI | 0.006 | 0.704 | 1.000 | -0.109 | 0.464 | 0.903 | 0.044 | 0.315 | 1.000 | -0.062 | 0.672 | 0.903 |
| Maze 5 ITI | 0.027 | 0.429 | 1.000 | -0.221 | 0.136 | 0.498 | 0.026 | 0.437 | 1.000 | 0.048 | 0.742 | 0.903 |

| Supplementary Table 4 cont..: Natural log transformed linear regression, and Kendall’s tau-b models for Maze intertap intervals compared to non-motor assessment scales in the OFF-levodopa state (n=25) | | | | | | | | | | | | |
| --- | --- | --- | --- | --- | --- | --- | --- | --- | --- | --- | --- | --- |
|  | HAM-D | | | | | | RBD-Q | | | | | |
|  |  | | | | | |  | | | | | |
|  | Linear regression – natural log (ln) transform | | | Kendall’s tau-b | | | Linear regression – natural log (ln) transform | | | Kendall’s tau-b | | |
|  | R^2^ | *p* value | BH *p*  value | CC | *p* value | BH  *p* value | R^2^ | *p* value | BH *p*  value | CC | *p* value | BH  *p* value |
| Maze 1 ITI | 0.012 | 0.616 | 1.000 | 0.051 | 0.725 | 0.903 | 0.007 | 0.698 | 1.000 | 0.104 | 0.480 | 0.903 |
| Maze 2 ITI | 0.003 | 0.791 | 1.000 | 0.024 | 0.870 | 0.903 | 0.014 | 0.260 | 1.000 | 0.187 | 0.204 | 0.509 |
| Maze 3 ITI | 0.003 | 0.813 | 1.000 | 0.010 | 0.944 | 0.903 | 0.011 | 0.624 | 1.000 | 0.111 | 0.451 | 0.903 |
| Maze 4 ITI | 0.037 | 0.367 | 1.000 | -0.010 | 0.944 | 0.903 | 0.002 | 0.826 | 1.000 | 0.014 | 0.925 | 0.903 |
| Maze 5 ITI | 0.016 | 0.555 | 1.000 | 0.106 | 0.467 | 0.903 | 0.001 | 0.911 | 1.000 | 0.007 | 0.962 | 0.903 |
| CC: correlation coefficient; BH: Benjamini-Hochberg corrected  Numbers in **bold** indicate significant p values after Benjamini-Hochberg (BH) correction | | | | | | | | | | | | |

| Supplementary Table 5: Wilcoxon signed rank test for PD participants (n=22) levodopa response on mazes | | | |
| --- | --- | --- | --- |
|  | Z | *p* value | BH corrected *p* value |
| Maze 1 | **-2.419** | **0.016** | **0.019** |
| Maze 2 | **-4.042** | **<0.001** | **<0.001** |
| Maze 3 | **-2.971** | **0.003** | **0.005** |
| Maze 4 | -0.568 | 0.570 | 0.570 |
| Maze 5 | **-4.107** | **<0.001** | **<0.001** |
| Numbers in **bold** indicate significant p values after Benjamini-Hochberg (BH) correction | | | |

| Supplementary Table 6: Linear regression model results for levodopa response for natural log transformed delta maze intertap intervals compared to delta UPDRS scores | | | | | | |
| --- | --- | --- | --- | --- | --- | --- |
|  | delta motor UPDRS | |  | delta total UPDRS | |  |
|  | R^2^ | *p* value | BH corrected *p* value | R^2^ | *p* value | BH corrected *p* value |
| Maze 1 delta ITI | 0.119 | 0.125 | 0.125 | 0.127 | 0.112 | 0.124 |
| Maze 2 delta ITI | **0.402** | **0.002** | **0.020** | **0.413** | **0.002** | **0.020** |
| Maze 3 delta ITI | **0.259** | **0.018** | **0.034** | **0.329** | **0.007** | **0.023** |
| Maze 4 delta ITI | **0.267** | **0.017** | **0.034** | **0.266** | **0.017** | **0.034** |
| Maze 5 delta ITI | **0.261** | **0.018** | **0.034** | **0.301** | **0.01** | **0.025** |
| Numbers in **bold** indicate significant p values after Benjamini-Hochberg (BH) correction | | | | | | |

| Supplementary Table 7: FOG vs noFOG subgroup analysis | | | | | | |
| --- | --- | --- | --- | --- | --- | --- |
|  | noFOG vs FOG (Mann-Whitney) | | | | | |
| Medication state: | OFF-levodopa | | | ON-levodopa | | |
|  | Z | *p* value | BH corrected *p* value | Z | *p* value | BH corrected *p* value |
| Maze 1 | -2.389 | 0.016 | **0.016** | -2.321 | 0.020 | **0.025** |
| Maze 2 | -2.525 | 0.010 | **0.017** | -2.456 | 0.014 | **0.023** |
| Maze 3 | -2.457 | 0.013 | **0.016** | -2.867 | 0.004 | **0.020** |
| Maze 4 | -2.867 | 0.003 | **0.015** | -2.116 | 0.034 | **0.034** |
| Maze 5 | -2.662 | 0.006 | **0.015** | -2.798 | 0.005 | **0.013** |
| Numbers in **bold** indicate significant p values after Benjamini-Hochberg (BH) correction | | | | | | |

| Supplementary Table 8: Levodopa response of the FOG and noFOG subgroups (Wilcoxon signed rank test) | | | | | | |
| --- | --- | --- | --- | --- | --- | --- |
| Group: | noFOG | | | FOG | | |
| Medication state: | OFF vs ON levodopa | | | OFF vs ON levodopa | | |
|  | Z | *p* value | BH corrected *p* value | Z | *p* value | BH corrected *p* value |
| Maze 1 | -2.166 | 0.030 | 0.051 | -1.260 | 0.208 | 0.519 |
| Maze 2 | **-3.296** | **0.001** | **0.005** | -2.380 | 0.017 | 0.086 |
| Maze 3 | **-3.107** | **0.002** | **0.005** | -1.260 | 0.208 | 0.346 |
| Maze 4 | -0.282 | 0.778 | 0.778 | -0.980 | 0.327 | 0.409 |
| Maze 5 | -1.475 | 0.140 | 0.175 | -0.280 | 0.779 | 0.779 |
| Numbers in **bold** indicate significant p values after Benjamini-Hochberg (BH) correction | | | | | | |

| Supplementary Table 9: Natural log transformed linear regression, and Kendall’s tau-b models for Maze intertap intervals (ITI) compared to spatiotemporal gait measures in PD participants in the ON levodopa state (n=21) | | | | | | | | | | | | |
| --- | --- | --- | --- | --- | --- | --- | --- | --- | --- | --- | --- | --- |
|  | Stride-length | | | | | | Stride-time | | | | | |
|  |  | | | | | |  | | | | | |
|  | Linear regression – natural log (ln) transform | | | Kendall’s tau-b | | | Linear regression – natural log (ln) transform | | | Kendall’s tau-b | | |
|  | R^2^ | *p* value | BH *p*  value | CC | *p* value | BH  *p* value | R^2^ | *p* value | BH *p*  value | CC | *p* value | BH  *p* value |
| Maze 1 ITI | 0.262 | **0.015** | **0.043** | -0.359 | **0.019** | **0.028** | 0.000 | 0.990 | 0.990 | -0.004 | 0.978 | 0.978 |
| Maze 2 ITI | 0.321 | **0.006** | **0.033** | -0.394 | **0.010** | **0.021** | 0.020 | 0.530 | 0.624 | 0.048 | 0.756 | 0.796 |
| Maze 3 ITI | 0.338 | **0.005** | **0.033** | -0.403 | **0.009** | **0.019** | 0.043 | 0.354 | 0.443 | 0.056 | 0.714 | 0.793 |
| Maze 4 ITI | 0.170 | 0.057 | 0.088 | -0.377 | **0.014** | **0.022** | 0.001 | 0.907 | 0.955 | -0.074 | 0.632 | 0.790 |
| Maze 5 ITI | 0.306 | **0.008** | **0.033** | -0.411 | **0.007** | **0.030** | 0.008 | 0.685 | 0.761 | 0.065 | 0.672 | 0.791 |

| Supplementary Table 9 cont..: Natural log transformed linear regression, and Kendall’s tau-b models for Maze intertap intervals (ITI) compared to spatiotemporal gait measures in PD participants in the ON levodopa state (n=21) | | | | | | | | | | | | |
| --- | --- | --- | --- | --- | --- | --- | --- | --- | --- | --- | --- | --- |
|  | Stride-width | | | | | | Stride-velocity | | | | | |
|  | Linear regression – natural log (ln) transform | | | Kendall’s tau-b | | | Linear regression – natural log (ln) transform | | | Kendall’s tau-b | | |
|  | R^2^ | *p* value | BH *p*  value | CC | *p* value | BH *p*  value | R^2^ | *p* value | BH *p*  value | CC | *p* value | BH  *p* value |
| Maze 1 ITI | **0.241** | **0.020** | **0.044** | **0.411** | **0.007** | **0.025** | 0.163 | 0.062 | 0.089 | **-0.411** | **0.007** | **0.018** |
| Maze 2 ITI | **0.384** | **0.002** | **0.020** | **0.411** | **0.007** | **0.021** | **0.252** | **0.017** | **0.043** | **-0.550** | **0.000** | **0.007** |
| Maze 3 ITI | **0.449** | **0.0006** | **0.013** | **0.524** | **0.001** | **0.004** | **0.291** | **0.010** | **0.033** | **-0.541** | **0.000** | **0.004** |
| Maze 4 ITI | 0.222 | 0.027 | 0.054 | **0.394** | **0.010** | **0.019** | 0.100 | 0.152 | 0.203 | **-0.394** | **0.010** | **0.017** |
| Maze 5 ITI | 0.190 | 0.042 | 0.070 | 0.307 | 0.045 | 0.060 | 0.222 | 0.027 | 0.054 | **-0.515** | **0.001** | **0.004** |
| CC: correlation coefficient; BH: Benjamini-Hochberg corrected  Numbers in **bold** indicate significant p values after Benjamini-Hochberg (BH) correction | | | | | | | | | | | | |

| Supplementary Table 10: Natural log transformed linear regression, and Kendall’s tau-b models for Maze intertap intervals (ITI) compared to standard PD assessment scales in PD participants in the ON levodopa state (n=21) | | | | | | | | | | | | |
| --- | --- | --- | --- | --- | --- | --- | --- | --- | --- | --- | --- | --- |
|  | Motor UPDRS | | | | | | Total UPDRS | | | | | |
|  | Linear regression – natural log (ln) transform | | | Kendall’s tau-b | | | Linear regression – natural log (ln) transform | | | Kendall’s tau-b | | |
|  | R^2^ | *p* value | BH *p*  value | CC | *p* value | BH *p*  value | R^2^ | *p* value | BH *p*  value | CC | *p* value | BH  *p* value |
| Maze 1 ITI | 0.130 | 0.108 | 0.135 | 0.220 | 0.164 | 0.479 | 0.143 | 0.090 | 0.129 | 0.177 | 0.264 | 0.479 |
| Maze 2 ITI | 0.259 | 0.019 | 0.080 | 0.297 | 0.061 | 0.479 | 0.289 | 0.012 | 0.080 | 0.253 | 0.109 | 0.479 |
| Maze 3 ITI | 0.265 | 0.017 | 0.080 | 0.325 | 0.040 | 0.479 | 0.315 | 0.008 | 0.080 | 0.339 | 0.032 | 0.479 |
| Maze 4 ITI | 0.107 | 0.147 | 0.153 | 0.239 | 0.131 | 0.479 | 0.112 | 0.138 | 0.153 | 0.224 | 0.156 | 0.479 |
| Maze 5 ITI | 0.252 | 0.020 | 0.080 | 0.316 | 0.046 | 0.479 | 0.261 | 0.018 | 0.080 | 0.310 | 0.050 | 0.479 |
| CC: correlation coefficient; BH: Benjamini-Hochberg corrected  Numbers in **bold** indicate significant p values after Benjamini-Hochberg (BH) correction | | | | | | | | | | | | |

| Supplementary Table 11: Natural log transformed linear regression, and Kendall’s tau-b models for Maze intertap intervals compared to non-motor assessment scales in the ON-levodopa state (n=21) | | | | | | | | | | | | |
| --- | --- | --- | --- | --- | --- | --- | --- | --- | --- | --- | --- | --- |
|  | MoCA | | | | | | HAM-A | | | | | |
|  |  | | | | | |  | | | | | |
|  | Linear regression – natural log (ln) transform | | | Kendall’s tau-b | | | Linear regression – natural log (ln) transform | | | Kendall’s tau-b | | |
|  | R^2^ | *p* value | BH *p*  value | CC | *p* value | BH  *p* value | R^2^ | *p* value | BH *p*  value | CC | *p* value | BH  *p* value |
| Maze 1 ITI | 0.120 | 0.114 | 0.570 | -0.169 | 0.290 | 0.573 | 0.000 | 0.965 | 1.000 | 0.009 | 0.955 | 0.796 |
| Maze 2 ITI | 0.269 | 0.013 | 0.240 | -0.352 | 0.028 | 0.357 | 0.006 | 0.732 | 1.000 | 0.063 | 0.690 | 0.727 |
| Maze 3 ITI | 0.277 | 0.012 | 0.240 | -0.361 | 0.024 | 0.357 | 0.023 | 0.498 | 1.000 | 0.153 | 0.333 | 0.573 |
| Maze 4 ITI | 0.070 | 0.235 | 0.940 | -0.178 | 0.265 | 0.573 | 0.000 | 0.950 | 1.000 | -0.009 | 0.955 | 0.796 |
| Maze 5 ITI | 0.227 | 0.025 | 0.240 | -0.343 | 0.032 | 0.357 | 0.001 | 0.892 | 1.000 | 0.027 | 0.864 | 0.763 |

| Supplementary Table 11 cont..: Natural log transformed linear regression, and Kendall’s tau-b models for Maze intertap intervals compared to non-motor assessment scales in the ON-levodopa state (n=21) | | | | | | | | | | | | |
| --- | --- | --- | --- | --- | --- | --- | --- | --- | --- | --- | --- | --- |
|  | HAM-D | | | | | | RBD-Q | | | | | |
|  |  | | | | | |  | | | | | |
|  | Linear regression – natural log (ln) transform | | | Kendall’s tau-b | | | Linear regression – natural log (ln) transform | | | Kendall’s tau-b | | |
|  | R^2^ | *p* value | BH *p*  value | CC | *p* value | BH  *p* value | R^2^ | *p* value | BH *p*  value | CC | *p* value | BH  *p* value |
| Maze 1 ITI | 0.015 | 0.595 | 1.000 | -0.076 | 0.630 | 0.727 | 0.019 | 0.545 | 1.000 | 0.126 | 0.426 | 0.639 |
| Maze 2 ITI | 0.000 | 0.950 | 1.000 | 0.004 | 0.977 | 0.796 | 0.057 | 0.284 | 0.947 | 0.252 | 0.111 | 0.418 |
| Maze 3 ITI | 0.007 | 0.714 | 1.000 | 0.120 | 0.444 | 0.639 | 0.038 | 0.385 | 1.000 | 0.207 | 0.191 | 0.573 |
| Maze 4 ITI | 0.045 | 0.357 | 1.000 | -0.067 | 0.671 | 0.727 | 0.010 | 0.666 | 1.000 | 0.117 | 0.460 | 0.639 |
| Maze 5 ITI | 0.002 | 0.833 | 1.000 | 0.040 | 0.799 | 0.749 | 0.037 | 0.390 | 1.000 | 0.198 | 0.211 | 0.573 |
| CC: correlation coefficient; BH: Benjamini-Hochberg corrected  Numbers in **bold** indicate significant p values after Benjamini-Hochberg (BH) correction | | | | | | | | | | | | |
